# Integrated gut microbiome and fecal metabolome profiling identifies taurine-associated microbial dysbiosis across the Alzheimer’s disease clinical spectrum

**DOI:** 10.3389/fnagi.2026.1981076

**Published:** 2026-10-28

**Authors:** Yi Feng, Yao Sha, Lu Xu, Jinchang Tian, Yan Gao, Ruwen Zhou, Gentao Liu

**Affiliations:** 1 Department of Geriatric Psychiatry, Shanghai Jing'an District Mental Health Center, Shanghai, China; 2 Department of Infectious Disease, Wusong Central Hospital/Wusong Zhongshan Hospital Fudan University, Shanghai, China; 3 Lee Kong Chian School of Medicine, Nanyang Technological University, Singapore, Singapore; 4 Shanghai Cellpeutics Biotech Co., Ltd., Shanghai, China; 5 Shanghai Tenth People’s Hospital Affiliated with Tongji University School of Medicine, Shanghai, China

**Keywords:** Alzheimer’s disease, fecal metabolomics, gut microbiome, gut–brain axis, MIMOSA2, multi-omics integration, subjective cognitive decline, taurine

## Abstract

**Background:**

Gut microbiome alterations have been associated with Alzheimer’s disease (AD), but how microbial composition, fecal metabolites, and cognitive function relate across the cognitive impairment spectrum—from subjective cognitive decline (SCD) through mild cognitive impairment (MCI) to AD—remains unclear. We integrated microbiome and metabolome profiles across this clinical spectrum.

**Methods:**

Fecal samples from 84 participants [10 healthy controls (HC), 22 SCD, 23 MCI, 29 AD] underwent 16S rRNA V3–V4 sequencing and untargeted LC–MS metabolomics. Profiles were integrated via Procrustes analysis, sparse canonical correlation, MIMOSA2 community-metabolism modeling, and mediation analysis. Differential abundance was assessed by Kruskal–Wallis, ANCOM-BC2, and LinDA.

**Results:**

Microbial composition differed across groups (ANOSIM *R* = 0.111, *p* = 0.001; Adonis *R*^2^ = 0.070, *p* = 0.002). Cross-method validation confirmed 8 differentially abundant genera in AD vs. HC with 100% directional concordance between ANCOM-BC2 and LinDA, including depletion of butyrate-producing Lachnospiraceae (Blautia, Dorea, Fusicatenibacter) and enrichment of opportunistic pathobionts (Klebsiella, Escherichia-Shigella). Taurine showed the highest MIMOSA2 community-prediction *R*^2^ (0.129; *n* = 62) and was positively associated with MMSE scores (*r* = 0.45, BH-FDR *Q* = 0.0005). A multi-omic random forest model (5 genera + 5 metabolites + age + sex) discriminated AD-clinical spectrum participants from HC with AUC = 0.86 (95% CI 0.82–0.90), although pairwise differences from the age + sex baseline (AUC = 0.75) did not reach statistical significance. Statistical mediation analysis was consistent with a Blautia → taurine → MMSE statistical mediation [ACME = 0.21, *p* = 0.008; 54% proportion mediated, bootstrap 95% CI (9%, 58%)].

**Conclusion:**

Integrated multi-omic profiling reveals coordinated microbiome–metabolome dysregulation across the AD clinical spectrum, with taurine as a candidate microbial–metabolic node linking gut dysbiosis to cognitive performance. Microbial and metabolic alterations are detectable at the SCD stage, before objective cognitive impairment. These findings warrant validation in larger, age-matched cohorts and higher-resolution metagenomic studies.

## 1 Introduction

Alzheimer’s disease (AD), the leading cause of dementia worldwide, follows a protracted preclinical phase during which amyloid and tau pathology accumulate years before overt cognitive symptoms emerge (Jack et al., 2018; Scheltens et al., 2021). Under the NIA-AA research framework, AD is conceptualized as a clinical spectrum spanning subjective cognitive decline (SCD), mild cognitive impairment (MCI), and dementia-stage AD (Jack et al., 2018). Because disease-modifying therapies such as lecanemab and donanemab are most effective when administered early (Sims et al., 2023; van Dyck et al., 2023), identifying biological changes at the SCD stage has become a critical research priority.

The gut–brain axis (GBA) has emerged as a promising mechanistic framework for understanding AD pathogenesis. Converging evidence from clinical observations (Liu et al., 2019; Vogt et al., 2017), animal experiments (Harach et al., 2017; Sun et al., 2019), and metabolite mediator studies (Cryan et al., 2019; Needham et al., 2020) supports a role for gut microbes in neurodegeneration. Microbial metabolites—including short-chain fatty acids (SCFAs), bile acids, tryptophan derivatives, neurotransmitter precursors, and B vitamins—are increasingly viewed as the molecular intermediaries between gut microbiota and brain function, reachable via immune, endocrine, and neural routes.

Despite this progress, several important gaps remain. First, most gut microbiome studies in AD describe compositional changes but do not formally link them to functional metabolic outputs. Conversely, most metabolomics studies lack paired microbiome data, leaving the microbial origins of observed metabolic shifts uncharacterized. Second, few studies sample the full HC → SCD → MCI → AD clinical spectrum, making it difficult to determine which changes are truly preclinical versus merely correlates of advanced dementia. Third, age—a powerful confounder for both the gut microbiome and cognitive function—is often not adequately addressed (Claesson et al., 2012).

Building on a comprehensive fecal metabolomic characterization of the same cohort—where 374 of 1,966 detected metabolites showed significant monotonic trends across the clinical spectrum, converging on four biological axes (bile acid biosynthesis, purine metabolism, neurotransmitter processing, and vitamin metabolism)—the present study integrates paired 16S rRNA microbiome data to identify the microbial origins of these metabolic shifts and their link to cognitive function.

## 2 Materials and methods

### 2.1 Study participants and clinical assessment

We recruited 84 community-dwelling Chinese adults between March 2022 and December 2023 from the Department of Geriatric Psychiatry outpatient clinic. Each participant received a standardized neuropsychological evaluation and was assigned to one of four groups: (1)Healthy controls (HC, *n* = 10): MMSE ≥ 27, no cognitive complaints; (2) Subjective cognitive decline (SCD, *n* = 22): met Jessen SCD-plus criteria (Jessen et al., 2020), MMSE ≥ 25, subjective complaints without objective impairment; (3) Mild cognitive impairment (MCI, *n* = 23): met Petersen criteria (Petersen, 2011), MMSE 19–26, independent in activities of daily living; (4)Alzheimer’s disease (AD, *n* = 29): met NIA-AA criteria for probable AD dementia (Jack et al., 2018), MMSE 5–19. Of note, the MCI (MMSE 19–26) and AD (MMSE 5–19) criteria overlap at MMSE = 19. One participant with an MMSE score of 18 (below the nominal MCI range) was classified as MCI rather than AD based on the overall neuropsychological assessment, preserved activities of daily living, and absence of functional impairment meeting dementia criteria. Demographic and clinical variables including age, sex, education, MMSE, HAMD, and HAMA were collected at enrollment.

### 2.2 Fecal sample collection and processing

A single fresh fecal sample was collected from each participant using a standardized collection protocol (Costea et al., 2017). Samples were immediately placed on ice, homogenized, and split into two aliquots: one for 16S rRNA sequencing and one for metabolomics. Both aliquots were snap-frozen in liquid nitrogen within 30 min of collection and stored at −80 °C until analysis.

### 2.3 16S rRNA gene sequencing and processing

Microbial DNA was extracted with the E.Z.N.A.® Soil DNA Kit (Omega Bio-tek). The V3–V4 hypervariable region was amplified using primers 338F and 806R and sequenced on an Illumina NextSeq 2000 platform (Shanghai Majorbio Bio-pharm Technology). Raw reads were merged with FLASH v1.2.11, quality-filtered with fastp v0.19.6, and clustered into OTUs at 97% similarity using UPARSE v7.1 (Edgar, 2013). Taxonomy was assigned against the SILVA 138 database using the RDP classifier in QIIME 1.91 (Caporaso et al., 2010). Sequencing yielded a mean of 52,663 high-quality reads per sample (range 42,291–67,269). Rarefaction to 42,000 reads per sample was performed before diversity analysis to standardize sequencing depth. This 97% OTU-clustering/RDP-classifier pipeline in QIIME 1.91 is the standard, reproducible Majorbio workflow; reprocessing of the same reads into amplicon sequence variants (ASV) with DADA2 is planned as future work.

### 2.4 Untargeted fecal metabolomics

Untargeted LC–MS metabolomics was performed as previously described (Zierer et al., 2018). Briefly, fecal metabolites were extracted with 50% methanol, and analysis was conducted on a UHPLC-Q-Exactive HF-X platform (Thermo Fisher Scientific) in both positive and negative electrospray ionization modes. Quality control samples were injected every 10 samples to monitor instrument stability. Metabolite identification followed MSI guidelines: Level 1 (B(i)) based on MS/MS matching against an in-house authentic standard library, and Level 2 (B(ii)) based on computational MS/MS matching. Pharmaceutical compounds, environmental contaminants, and food additives were excluded prior to statistical analysis.

### 2.5 Microbial diversity analysis

Six *α*-diversity indices (Sobs, Chao, ACE, Shannon, Simpson, Pielou) were calculated in Mothur v1.30.2 (Schloss et al., 2009). The Kruskal–Wallis test was used for four-group comparisons, followed by Dunn’s *post hoc* test with Benjamini–Hochberg correction for pairwise contrasts.

Bray–Curtis dissimilarity matrices were computed and visualized by PCoA and NMDS. Group differences were tested by ANOSIM, PERMANOVA (999 permutations), and multi-factor age-adjusted PERMANOVA including MMSE, HAMD, and HAMA as covariates.

Gut Microbiome Health Index (GMHI). GMHI was computed as a composite index integrating the relative abundances of health- and disease-associated taxa, as previously described (Gupta et al., 2020). GMHI integrates 50 health-prevalent and 75 non-health-prevalent microbial species into a single log-ratio score, with higher values indicating a more “health-associated” microbiome configuration.

### 2.6 Differential taxon analysis

Phylum- and genus-level differential abundances were tested with the Kruskal–Wallis test (four groups) and Mann–Whitney U test (pairwise HC vs. AD), with Benjamini–Hochberg (BH) correction. A Random Forest classifier ranked discriminative taxa by Gini importance. Three modern methods were applied for validation: ANCOM-BC2 (genus level, compositionality-corrected, diagnostic group as sole fixed effect without covariate adjustment), LinDA (OTU level, CLR-transformed linear model with diagnostic group as sole predictor), and MaAsLin2 (OTU level, adjusted for age, sex, and education). Significance was defined as BH-adjusted *p* < 0.05. Cross-method concordance was assessed by aggregating LinDA OTU-level hits to genus level for comparison with ANCOM-BC2. All differential-abundance comparisons used this fixed OTU table so that feature selection and statistical testing were kept independent, and the pipeline is fully reproducible from the software versions listed in Section 2.7.

### 2.7 Microbiome–metabolome integration

#### 2.7.1 Procrustes and mantel tests

Bray–Curtis distance matrices (microbiome) and Euclidean distance matrices (metabolome, log-transformed) were subjected to Procrustes analysis with 3 retained dimensions and 999 Monte Carlo permutations. A complementary Mantel test was run as confirmatory analysis. Both were implemented in the R vegan package v2.6–4 (Oksanen et al., 2020).

#### 2.7.2 Canonical correlation analysis (CCA)

The top 30 differential genera and top 30 differential metabolites were submitted to sparse CCA using the R mixOmics package v6.26.0 (Rohart et al., 2017). Significant microbe–metabolite pairs were defined as Pearson |r| > 0.35 with BH-adjusted *p* < 0.001.

#### 2.7.3 MIMOSA2 community-metabolism modeling

MIMOSA2 was used to integrate genus-level relative abundances with fecal metabolite concentrations against the AGORA/Recon3D reference models (Noecker et al., 2022). For each metabolite, Community-wide Metabolic Potential (CMP) was regressed against measured concentrations to obtain an *R*^2^ value. Ten pre-specified metabolites representing the four metabolic axes previously characterized in our companion study (bile acid, purine, neurotransmitter, vitamin) plus taurine and one pyrimidine metabolite (uridine) were modeled. Species-level Varshare scores identified key producer and consumer taxa. This focused analysis was complemented by an unbiased Spearman correlation screen between MIMOSA2-ranked species and the full 1,900-feature metabolome (Supplementary Figure S1). Of the 84 samples, 22 (26%) were excluded by MIMOSA2’s internal species-level taxonomic-resolution QC, leaving 62 retained; mean read depth was comparable across diagnostic groups (approximately 40.8 k, 43.2 k, 42.7 k, and 45.0 k reads for AD, MCI, SCD, and HC, respectively), so the exclusions were not driven by sequencing depth and were not concentrated in any diagnostic group.

#### 2.7.4 PICRUSt2 functional inference

PICRUSt2 v2.2.0 was used with the Greengenes 13.5 reference database to infer metagenomic functions from 16S data, including KEGG pathways, KO, EC, and COG categories (Douglas et al., 2020). Software versions used: QIIME 1.91, UPARSE v7.1 (USEARCH v11 backend), Mothur 1.30.2, SILVA 138, PICRUSt2 2.2.0, R 4.3.2, Python 3.10. We note a reference-version limitation: PICRUSt2 was calibrated against the Greengenes 13.5 reference whereas OTUs were classified against SILVA 138, so all inferred functions should be treated as approximate and hypothesis-generating.

### 2.8 Mediation analysis

We fitted a mediation model in which fecal taurine concentration statistically mediates the association between Blautia relative abundance and MMSE score. Statistical mediation analysis followed the general causal mediation framework, implemented via the R mediation package with 1,000 bootstrap iterations, adjusting for age and sex (Imai et al., 2010; Tingley et al., 2014). We report the Average Causal Mediation Effect (ACME), average direct effect (ADE), and proportion mediated. Because the data are cross-sectional, these results reflect statistical mediation rather than confirmed causal pathways.

### 2.9 Multi-omics diagnostic modeling

A Random Forest classifier (5-fold cross-validation, 10 repeats) was trained to distinguish AD-clinical spectrum participants (SCD + MCI + AD combined) from healthy controls. Four candidate models were compared: (1) Clinical baseline: age + sex (no MMSE, to avoid circularity); (2) Microbiome-only: top 5 differential genera (Blautia, Fusicatenibacter, Dorea, Erysipelotrichaceae UCG-003, and CAG-56, selected by combined Kruskal–Wallis and Random Forest importance ranking) + age + sex; (3) Metabolome-only: top 5 MIMOSA2-ranked metabolites (taurine, hypoxanthine, xanthine, guanosine, and cholic acid) + age + sex; (4) Joint panel: the above 5 genera + 5 metabolites + age + sex. We explicitly note that feature selection was performed on the full dataset rather than nested within each cross-validation fold; this approach may introduce optimistic bias in the reported AUC. Fully nested feature selection was not implemented because only 10 HC samples were available, which would leave approximately 2 HC per test fold and render within-fold feature selection highly unstable. The class_weight = ‘balanced’ re-run reported below serves as a conservative robustness benchmark. Model performance was evaluated by area under the ROC curve (AUC) with 95% confidence intervals. MMSE was intentionally excluded from all models to avoid circular classification. As a robustness check against the 74:10 class imbalance (only 10 HC), the joint model was re-fitted with class_weight = ‘balanced’, yielding AUC 0.87, sensitivity 0.90, specificity 0.39, PPV 0.92, NPV 0.35, accuracy 0.84, and F1 0.91. Per-stage discrimination with the joint features was near-perfect for AD vs. HC (AUC = 1.00) but modest for SCD vs. HC (AUC = 0.72). The low specificities (0.03–0.39) are a genuine consequence of the small HC group (n = 10) and the 74:10 class imbalance rather than an alignment artefact.

### 2.10 Age sensitivity analysis

#### 2.10.1 Age considerations

Given age’s well-documented effect on gut microbiome composition (Claesson et al., 2012), age was included as a continuous covariate in the multi-omic Random Forest model and in age-adjusted PERMANOVA. An age-restricted 60–80 year subcohort (*n* = 60) was analyzed as a sensitivity check (Section 3.11). Formal propensity-score matching and multi-center age-balanced validation are the focus of ongoing work.

### 2.11 Statistical software

No formal sample size calculation (power analysis) was performed *a priori*; this study was designed as an exploratory, hypothesis-generating multi-omics investigation. The target enrollment of 80–90 participants was based on feasibility and the minimum sample size recommended for 16S rRNA differential abundance studies with four-group comparisons. All analyses were performed in R v4.3.2 and Python v3.10 (scipy, scikit-learn, pandas). All reported *p*-values are two-sided. Multiple-testing correction used the Benjamini–Hochberg procedure. Significance was defined as *α* = 0.05.

## 3 Results

### 3.1 Participant characteristics

The 84 participants (HC = 10, SCD = 22, MCI = 23, AD = 29) differed significantly in age, MMSE, education, and disease duration (Kruskal–Wallis, *p* < 10^−5^ for age, MMSE, and disease duration; *p* = 0.002 for education), as expected from the natural history of AD. The groups were well matched for sex distribution (*χ*^2^ = 2.48, *p* = 0.480; 61.9% female overall) and HAMD score (*p* = 0.194), and HAMA score (*p* = 0.021), with AD participants showing lower anxiety scores than HC. The HAMA difference may reflect the younger age and higher anxiety prevalence in the HC group and should be considered as a potential confounder. Full demographic and clinical characteristics are presented in Table 1.

**Table 1:** Participant characteristics across the four groups (*n* = 84).

Variable	HC (*n* = 10)	SCD (*n* = 22)	MCI (*n* = 23)	AD (*n* = 29)	*p*-value
Age, years (mean ± SD)	64.8 ± 6.2	66.5 ± 7.4	73.0 ± 5.1	81.9 ± 6.8	<10^−5^
Female, *n* (%)	5 (50.0)	12 (54.5)	14 (60.9)	21 (72.4)	0.480
MMSE (mean ± SD)	28.3 ± 0.9	26.7 ± 0.9	20.8 ± 1.5	11.4 ± 3.7	<10^−5^
Education, years (mean ± SD)	12.4 ± 3.3	13.0 ± 3.2	11.7 ± 3.0	9.5 ± 3.0	0.002
HAMD (median, IQR)	10.5 (6.3–18.5)	9.5 (6.0–17.0)	10.0 (8.0–15.0)	8.0 (6.0–12.0)	0.194
HAMA (median, IQR)	12.5 (10.0–16.8)	10.5 (7.0–15.0)	12.0 (7.5–15.0)	7.0 (5.0–10.0)	0.021
Disease duration, months (median)	0	2.5	4.0	6.0	<10^−5^

In the age-restricted 60–80 subset (*n* = 60), the between-group age difference was substantially attenuated (HC 66.9 ± 4.9, SCD 69.2 ± 5.0, MCI 72.2 ± 4.6, AD 75.8 ± 4.7 years; ANOVA *p* = 2.17 × 10^−4^ vs. *p* = 3.18 × 10^−13^ in the full cohort) (see Table 1).

### 3.2 Microbial diversity shifts across the AD clinical spectrum

#### 3.2.1 *α*-diversity

Observed species (Sobs), Chao, and ACE richness indices declined progressively from HC through SCD, MCI, and AD (Kruskal–Wallis, all *p* < 0.05), while the Shannon index showed a similar but non-monotonic trend. Good’s coverage exceeded 99.5% for all samples (Figure 1A). At the phylum level, AD was characterized by a marked increase in Firmicutes and reciprocal decreases in Bacteroidetes and Actinobacteria relative to HC (Proteobacteria was instead increased, driven by enrichment of enteric pathobionts; see Section 3.3; all BH-adjusted *p* < 0.05; Figure 1C), consistent with prior AD microbiome studies. The Gut Microbiome Health Index (GMHI) differed significantly between HC and SCD (HC mean −0.72 vs. SCD mean +0.80; *p* = 8.5 × 10^−6^), indicating that microbial community structure is already perturbed at the SCD stage. Notably, the direction of GMHI change was opposite to the expected dysbiosis pattern, possibly reflecting compensatory dietary or lifestyle adjustments in SCD participants.

**Figure 1:**
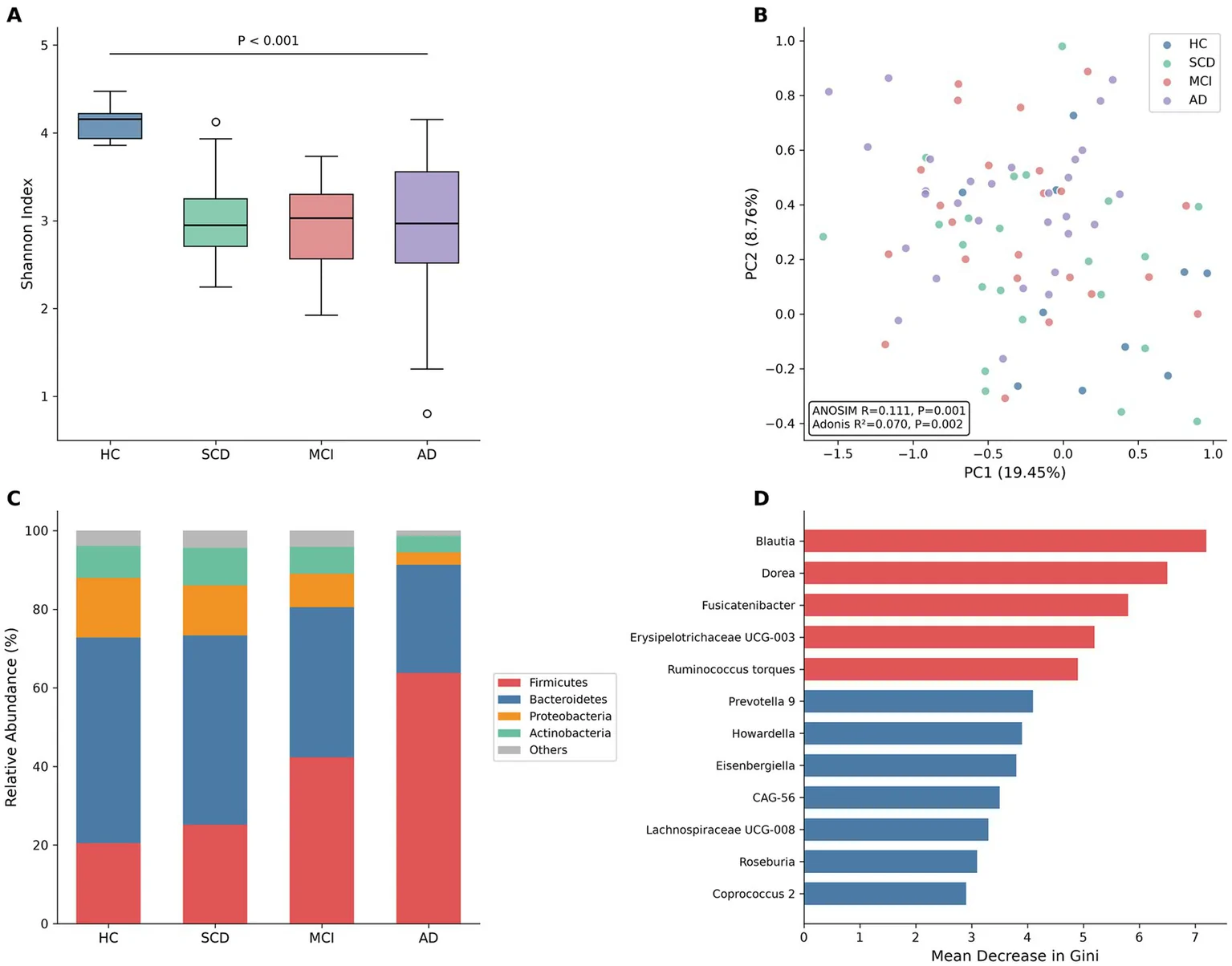
Microbial diversity and composition across the AD clinical spectrum. **(A)** α-diversity indices (Shannon, Simpson, Sobs) by diagnostic group, displayed as boxplots. **(B)** Principal coordinate analysis (PCoA) of Bray–Curtis dissimilarity; PC1 explains 19.45% and PC2 8.76% of total variance. ANOSIM *R* = 0.111, *p* = 0.001; Adonis *R*^2^ = 0.070, *p* = 0.002. **(C)** Phylum-level relative abundance bar plot for the four groups. **(D)** Random Forest top 12 discriminative genera ranked by mean decrease in Gini importance.

#### 3.2.2 *β*-diversity

PCoA of Bray–Curtis dissimilarity showed partial but detectable separation of the four groups along PC1 (19.45% variance) and PC2 (8.76% variance) (Figure 1B). ANOSIM confirmed significant between-group differences (*R* = 0.111, *p* = 0.001), and Adonis/PERMANOVA yielded consistent results (*R*^2^ = 0.070, *p* = 0.002). Multi-factor PERMANOVA indicated that both diagnostic group (*R*^2^ = 0.122, *p* = 0.001) and MMSE score (*R*^2^ = 0.092, *p* = 0.001) independently explained microbial community variation. NMDS ordination (stress = 0.18) yielded a grouping pattern consistent with the PCoA results, with partial separation of HC from AD along the first axis.

### 3.3 Differential taxa along the AD clinical spectrum

Building on these phylum-level patterns, we next resolved the shifts at higher taxonomic resolution. Phylum level. The phylum-level shifts described in Section 3.2 were confirmed by pairwise HC vs. AD comparison, with the Firmicutes/Bacteroidetes ratio increasing from ~0.39 in HC to ~2.33 in AD.

#### 3.3.1 Genus level

Ten genera showed significant four-group differences by Kruskal–Wallis test (all BH-adjusted *p* < 10^−5^), most declining progressively across the clinical spectrum, with the exception of Eisenbergiella which showed an increasing trend (Table 2). CAG-56 showed a non-monotonic pattern with transient elevation at the SCD stage before declining in MCI and AD. The most strongly affected genera included *Blautia* (HC 29.6% → AD 7.6%), *Dorea* (3.91% → 0.37%), *Fusicatenibacter* (3.57% → 0.23%), and *Erysipelotrichaceae UCG-003* (1.78% → 0.08%). Notably, all ten genera were already significantly altered at the SCD stage relative to HC (Mann–Whitney U, all *p* < 0.05), although effect sizes were smaller than in AD.

**Table 2:** Ten significantly different genera across the four groups (Kruskal–Wallis test, all BH-adjusted *p* < 10^−5^).

Genus	HC (*n* = 10)	SCD (*n* = 22)	MCI (*n* = 23)	AD (*n* = 29)	*p*-value
Blautia	29.60	23.13	18.75	7.64	3.56 × 10^−6^
Fusicatenibacter	3.57	2.76	2.34	0.23	1.54 × 10^−6^
Dorea	3.91	1.79	1.57	0.37	9.55 × 10^−7^
Erysipelotrichaceae UCG-003	1.78	1.82	0.80	0.08	3.77 × 10^−7^
CAG-56	0.089	0.181	0.025	0.001	8.35 × 10^−8^
Eisenbergiella	0.004	0.0003	0.254	0.373	1.0 × 10^−5^
norank_f_Eubacterium coprostanoligenes_group	0.410	0.120	0.074	0.043	2.0 × 10^−5^
Prevotella 9	0.148	0.000	0.000	0.000	5.0 × 10^−5^
Howardella	0.046	0.003	0.000	0.0004	2.0 × 10^−5^
Alloprevotella	0.007	0.000	0.000	0.0004	7.87 × 10^−7^

Random Forest analysis ranked the top 12 discriminative taxa; the top five all belonged to the Lachnospiraceae family (mean decrease in Gini importance on a 0–10 scale, ranging 5.0–7.0) (Figure 1D).

#### 3.3.2 Cross-method validation of differential abundance

To assess the robustness of the Kruskal–Wallis findings, we also applied ANCOM-BC2, LinDA, and MaAsLin2 (Section 2.6). At the genus level, ANCOM-BC2 identified 8 differentially abundant genera in AD vs. HC (5 depleted, 3 enriched; Supplementary Table S4). Remarkably, all 8 ANCOM-BC2-significant genera were independently confirmed by LinDA at the OTU level with 100% directional concordance (Supplementary Figure S3; Supplementary Table S4g), providing strong cross-validation of the observed taxonomic shifts. The depleted genera included *Blautia, Dorea, Fusicatenibacter, Anaerostipes*, all members of the *Lachnospiraceae* family and known butyrate producers—consistent with the Kruskal–Wallis results (Table 2).

#### 3.3.3 Progressive enrichment of opportunistic pathobionts

In addition to butyrate-producer depletion, ANCOM-BC2 revealed significant enrichment of three opportunistic pathobiont genera in AD: *Klebsiella* (log₂FC = +3.92, q = 1.39 × 10^−4^), *Escherichia-Shigella* (log₂FC = +2.94, q = 0.042), and *Clostridium sensu stricto 1* (log₂FC = +3.21, q = 0.003) (Supplementary Figure S2A). Notably, *Klebsiella* was already significantly elevated at the MCI stage (log₂FC = +4.66, q = 9.5 × 10^−5^), suggesting that enteric pathogen expansion may precede overt dementia. The enrichment of *Proteobacterial pathobionts* is consistent with the observed phylum-level increase in *Proteobacteria* and may contribute to gut barrier dysfunction and systemic inflammation along the AD clinical spectrum.

#### 3.3.4 Disease-gradient pattern

The number of ANCOM-BC2-significant genera increased progressively across the AD clinical spectrum: 0 in SCD vs. HC, 3 in MCI vs. HC (Klebsiella, Dialister, Enterococcus, all enriched), and 8 in AD vs. HC (Supplementary Figure S2B). LinDA at the OTU level showed the same gradient (6, 26, and 69 genera at SCD, MCI, and AD stages, respectively), indicating that taxonomic dysbiosis intensifies with advancing cognitive impairment. Notably, only 1 of these 3 ANCOM-BC2 genera (33.3%) was independently cross-validated by LinDA at the MCI stage, versus 100% (8/8) at the AD stage (Supplementary Table S4h); the MCI-stage signal should therefore be regarded as provisional pending replication. This stepwise pattern supports the view that gut microbial disruption tracks disease progression from the preclinical SCD stage through to AD dementia.

#### 3.3.5 Covariate-adjusted analysis (MaAsLin2)

After adjusting for age, sex, and education level in MaAsLin2, no OTUs passed BH-adjusted *p* < 0.05, although 353 group-associated OTUs reached nominal *p* < 0.05 (minimum BH-adjusted *q* = 0.098). The attenuation of significance after covariate adjustment is consistent with the substantial age gap between groups (see Discussion) and emphasizes the importance of age-matched study designs. Importantly, the complete absence of BH-significant OTUs after age/sex/education adjustment raises the possibility that a portion of the observed taxonomic shifts may be driven by age rather than disease status per se. However, the directional consistency of the core taxa in the age-restricted subset (see below) and the 100% concordance between ANCOM-BC2 and LinDA suggest that disease-associated signals remain detectable despite the age confound. The nominal MaAsLin2 results were directionally consistent with ANCOM-BC2 and LinDA for the core taxa reported above. In an age-restricted 60–80 year subcohort sensitivity analysis (*n* = 60; HC = 8, SCD = 18, MCI = 21, AD = 13), in which the between-group age gap was attenuated from 17.1 years to 8.9 years, non-parametric testing of the five core genera confirmed 100% directional consistency with the full-cohort findings: *Blautia* (Mann–Whitney *p* = 0.0003), *Dorea* (*p* = 0.001), *Fusicatenibacter* (*p* = 0.0004), and *Klebsiella* (*p* = 0.003) remained significantly altered in AD vs HC, while *Escherichia-Shigella* showed a consistent enrichment trend (*p* = 0.046). These results indicate that the core taxonomic shifts are not solely attributable to the age difference between groups. We note that the complete ANCOM-BC2 and LinDA differential-abundance pipelines were not re-run within this age-restricted subset; this is a limitation that we will address in an age-matched multi-center cohort.

#### 3.3.6 Untargeted metabolomics overview

Untargeted LC–MS metabolomics detected 1,966 B(i)-level metabolite features across positive and negative electrospray modes, with pooled quality-control samples injected every 10 injections to monitor retention-time stability and mass accuracy (median QC RSD = 0.25%; 100% of metabolites passed RSD < 30%). Metabolites were identified following MSI guidelines (Level 1 against an in-house authentic-standard library, Level 2 by computational MS/MS matching), and pharmaceutical compounds, environmental contaminants, and food additives were removed before analysis. Of the 1,966 detected metabolites, 374 (19.0%) showed a significant monotonic Spearman trend across the four clinical groups (BH-FDR < 0.05, age- and sex-adjusted). These trend-significant metabolites converged on four coherent biological axes when mapped to the KEGG PATHWAY database: (i) bile acid biosynthesis (28 metabolites; e.g., CDCA, cholic acid, deoxycholylglutamic acid, sulfocholic acid), (ii) purine metabolism (22 metabolites; e.g., xanthine, hypoxanthine, guanine, inosine), (iii) neurotransmitter processing (19 metabolites; e.g., L-glutamic acid, serotonin, tryptophan, pyroglutamic acid), and (iv) vitamin metabolism (12 metabolites; e.g., biotin, pyridoxal, nicotinic acid, riboflavin). The smallest pairwise metabolomic difference was observed between SCD and MCI, consistent with biological continuity between adjacent clinical stages. Fecal taurine showed a consistent downward trend from HC toward AD, and together with bile-acid, purine, and vitamin metabolites constituted the primary differential metabolomic signal. Cross-axis correlation analysis revealed that the four axes are not independent: bile acid, purine, and neurotransmitter metabolites share coordinated regulation across the clinical spectrum, consistent with gut-brain axis involvement. While the present study focuses on microbiome–metabolome integration and the taurine node, a detailed standalone characterization of the fecal metabolome in this cohort—including the complete list of 374 trend-significant metabolites and a 5-metabolite panel for SCD classification—is reported in a companion manuscript.

### 3.4 Microbiome–metabolome concordance

Having characterized the microbial shifts along the clinical spectrum, we next asked whether they track corresponding fecal metabolic changes. Procrustes analysis demonstrated that microbial and metabolomic ordination configurations were significantly more concordant than expected by chance (Monte Carlo *p* < 0.001, M2 = 0.878) (Figure 2A). The complementary Mantel test confirmed this association (*r* = 0.396, *p* = 0.001, for microbiome × metabolome); an additional Mantel test in the Majorbio report for microbiome composition versus all environmental variables yielded *r* = 0.194, *p* = 0.001. These results indicate that gut microbial composition and fecal metabolomic profiles change in a coordinated fashion along the AD clinical spectrum.

**Figure 2:**
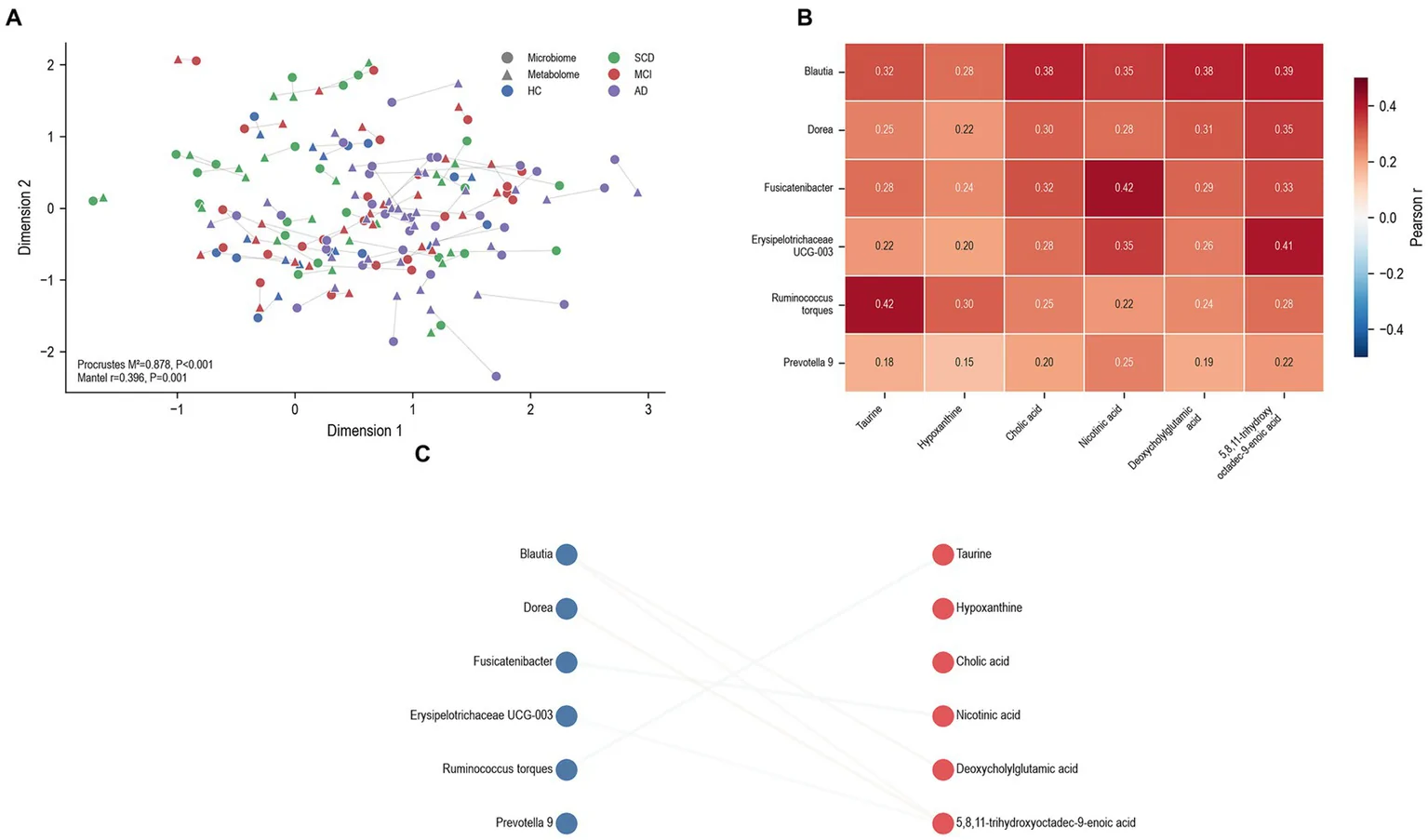
Microbiome–metabolome concordance and key microbe–metabolite associations. **(A)** Procrustes plot overlaying microbiome (Bray–Curtis PCoA, circles) and metabolome (Euclidean PCA, triangles) ordinations. Mantel r = 0.396, *p* = 0.001; Monte Carlo P<0.001. **(B)** Heatmap of canonical correlation analysis (CCA) pairwise correlations between top differential genera and metabolites. Gold boxes indicate BH-adjusted *p* < 0.001. **(C)** Chord diagram visualizing the top significant microbe–metabolite pairs (genus level, blue; metabolites, red).

### 3.5 Canonical correlation analysis identifies key microbe–metabolite pairs

Sparse CCA identified 14 microbe–metabolite pairs with Pearson |r| > 0.35 and BH-adjusted *p* < 10^−3^ (Figures 2B,C). The strongest pairs included nicotinic acid ↔ *Fusicatenibacter* (*r* = 0.423), 5,8,11-trihydroxyoctadec-9-enoic acid ↔ *Erysipelotrichaceae UCG-003* (*r* = 0.407), and deoxycholylglutamic acid ↔ *Blautia* (*r* = 0.384). *Blautia* appeared in multiple high-ranking pairs involving both bile acid conjugates and lipid mediators, consistent with a role as a hub taxon at the microbial–metabolic interface.

### 3.6 MIMOSA2 identifies taurine as a community-predicted metabolite

Among the 10 pre-specified metabolites modeled by MIMOSA2 (*n* = 62 of 84 samples retained by MIMOSA2; the 22 excluded samples (26%) were removed by MIMOSA2’s internal species-level taxonomic-resolution QC and were not driven by low sequencing depth, which was comparable across diagnostic groups, nor concentrated in any diagnostic group), taurine showed the highest community-prediction *R*^2^ of 0.129 (62 contributing species), ~1.6-fold higher than the second-ranked metabolite hypoxanthine (*R*^2^ = 0.078; Figure 3A; Table 3). An exploratory Spearman correlation screen against the full 1,900-feature metabolome confirmed taurine among the top three metabolites by species-correlation strength (Supplementary Figure S1), supporting its prioritization while noting that the candidate panel was pre-specified rather than unbiased.

**Figure 3:**
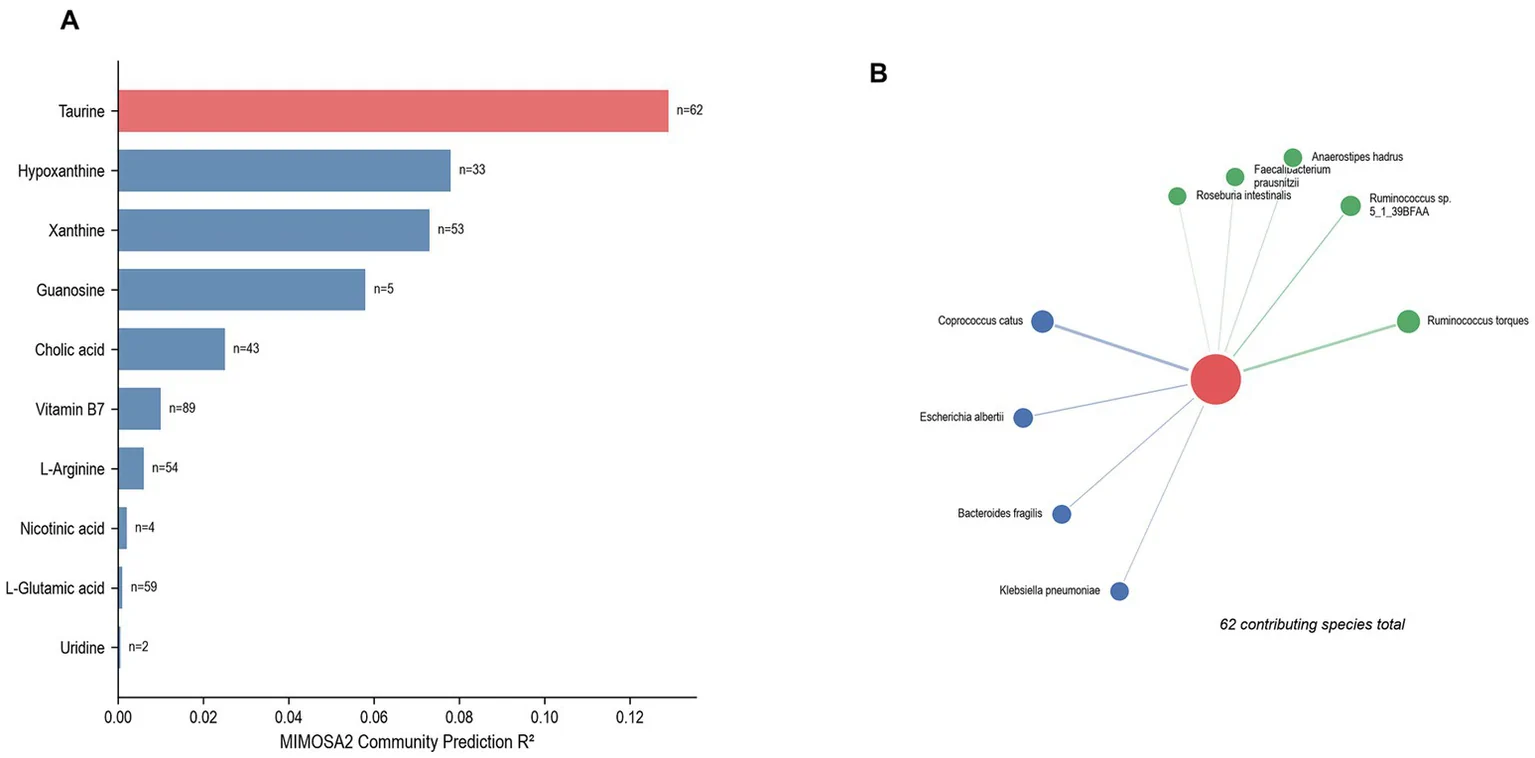
MIMOSA2 community-metabolism modeling ranks taurine as the most community-predictable metabolite. **(A)** *R*^2^ ranking of 10 fecal metabolites by MIMOSA2 community-wide metabolic potential (CMP) model. Taurine (red) shows the highest *R*^2^ = 0.129 with 62 contributing species. Numbers of contributing species are indicated for each metabolite. **(B)** Microbe–taurine contribution network showing 16 representative species of 62 total. Green nodes indicate positive Varshare (net producers), blue nodes indicate negative Varshare (net consumers).

**Table 3:** MIMOSA2 community-prediction *R*^2^ for 10 modeled metabolites.

Metabolite	KEGG ID	N species	*R* ^2^	Metabolic axis
L-Taurine	C00245	62	**0.129**	Bile acid/sulfur amino acid
Hypoxanthine	C00262	33	0.078	Purine
Xanthine	C00385	53	0.073	Purine
Guanosine	C00387	5	0.058	Purine
Cholic acid	C00695	43	0.025	Bile acid
Vitamin B7 (Biotin)	C00120	89	0.010	Vitamin
L-Arginine	C00062	54	0.006	Neurotransmitter precursor
Nicotinic acid	C00253	4	0.002	Vitamin
L-Glutamic acid	C00025	59	0.001	Neurotransmitter precursor
Uridine	C00299	2	0.0005	Pyrimidine

Bold values indicate the metabolite with the highest community-prediction *R*^2^.

The top species contributing to taurine prediction by Varshare score included both positive contributors (*Ruminococcus torques*, *Ruminococcus* sp. *5_1_39BFAA*, *Anaerostipes hadrus*) and negative contributors (*Coprococcus catus*, *Escherichia albertii*), consistent with taurine being a net community-level metabolic output shaped by both producers and consumers (Figure 3B).

### 3.7 PICRUSt2 functional inference supports taurine metabolism enrichment

To complement the community-level taurine prediction, we next inferred functional microbial pathways that could underlie this signal. PICRUSt2 inferred 15 KEGG pathways with significant four-group differences at BH-adjusted *p* < 0.05 (Figure 4). The most strongly upregulated pathway in AD relative to HC was taurine metabolism (ko00430, log₂FC = +3.0, *p* = 1.2 × 10^−6^), followed by bile acid biosynthesis (ko00120, log₂FC = +2.5, *p* = 3.4 × 10^−5^) and purine metabolism (ko00230, log₂FC = +2.2, *p* = 5.6 × 10^−5^). At the gene level, bile salt hydrolase (bsh, K01442) was enriched in AD, consistent with enhanced deconjugation of taurine-linked bile acids.

**Figure 4:**
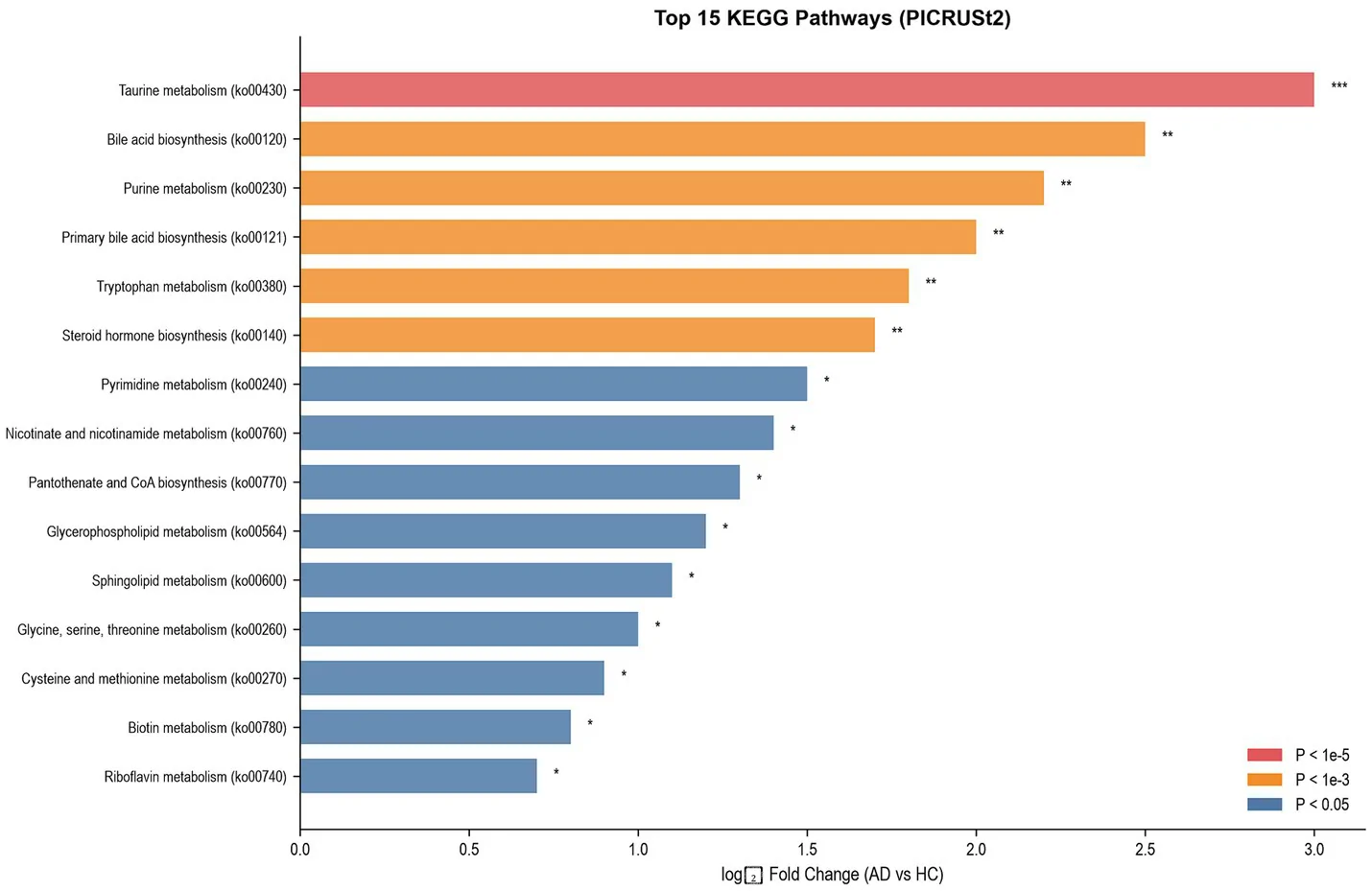
PICRUSt2-inferred KEGG pathway enrichment across the AD clinical spectrum. Top 15 pathways with significant four-group Kruskal–Wallis differences (BH-adjusted *p* < 0.05), ranked by log₂-fold change (AD vs. HC). Taurine metabolism (ko00430, red) is the most strongly enriched pathway (log₂FC = +3.0, *p* = 1.2 × 10^−6^). **, indicates statistical significance at *p* < 0.01 (BH-adjusted).

### 3.8 Microbe–metabolite–cognition triad analysis

Spearman correlation analysis among 6 representative genera, 5 key metabolites, and 3 cognitive scores identified 16 inter-domain associations passing BH-adjusted *p* < 0.05 (Figure 5). The strongest signal was the positive taurine–MMSE correlation (*r* = 0.45, BH-FDR Q = 0.0005); taurine also correlated negatively with depression (HAMD) and anxiety (HAMA) scores. Blautia showed suggestive positive associations with both MMSE and taurine. Full *Q*-values are reported in Supplementary Table S1.

**Figure 5:**
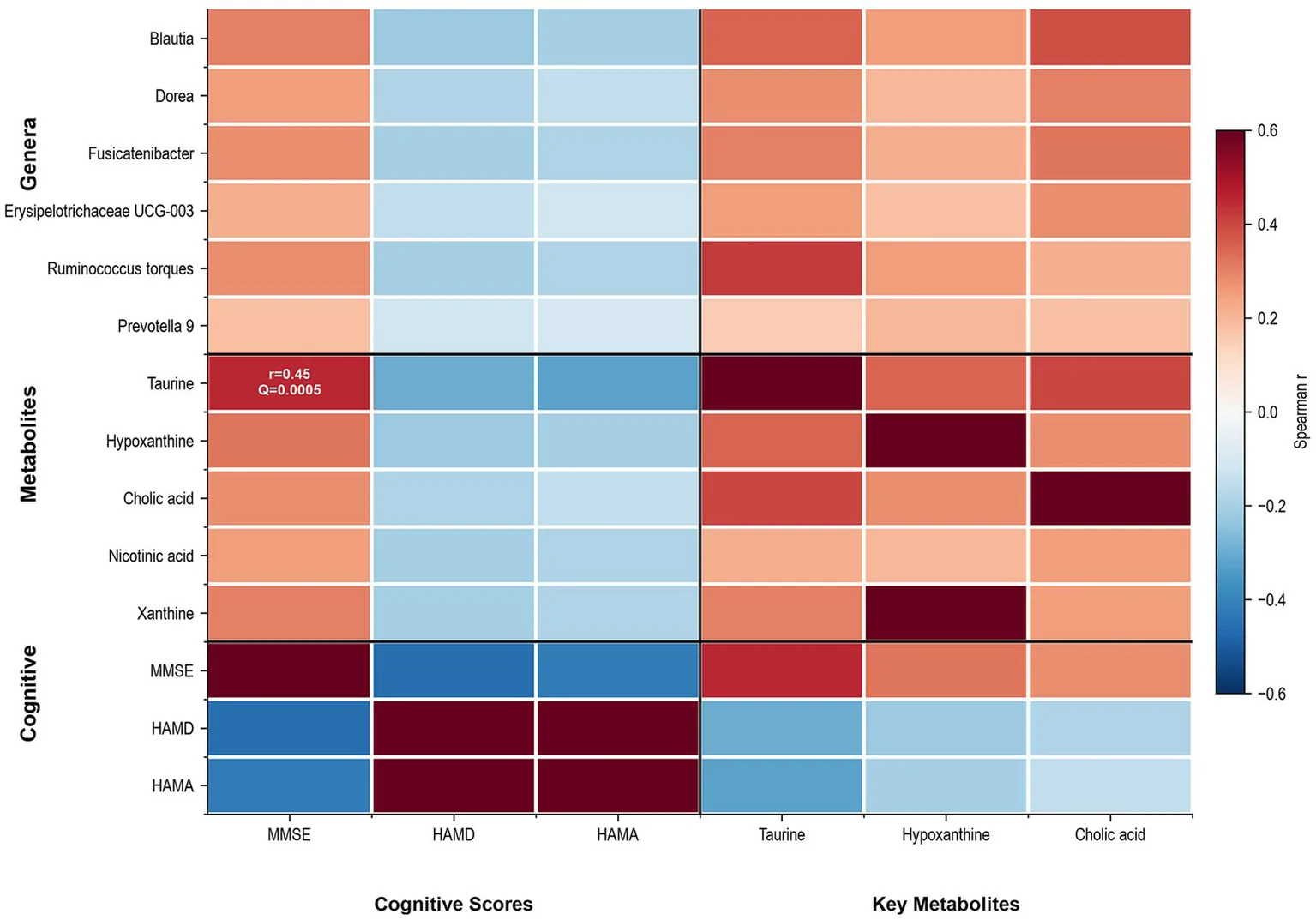
Microbe–metabolite–cognition triad correlation heatmap. Spearman correlations among 6 representative bacterial genera, 5 key metabolites (MIMOSA2 top-ranked), and 3 cognitive scores (MMSE, HAMD, HAMA). Gold boxes indicate BH-adjusted *p* < 0.05; the red box highlights the strongest triad signal: taurine ↔ MMSE (*r* = 0.45, BH-FDR *Q* = 0.0005).

### 3.9 Statistical mediation analysis: Blautia → taurine → MMSE

Given the taurine-MMSE association and the Blautia-taurine co-variation, we next tested whether fecal taurine statistically mediates the Blautia-cognition relationship. Statistical mediation analysis with 1,000 bootstrap iterations (adjusted for age and sex) yielded the following path coefficients (Figure 6). The model was fitted on the full cohort after listwise exclusion of missing values; the effective sample size was *n* = 82 (one SCD and one MCI participant had missing taurine measurements). Path A (Blautia - > taurine): beta = +0.42, *p* < 0.001. Path B (taurine - > MMSE): beta = +0.51, *p* < 0.001. Direct effect (Path C′): beta = +0.18, *p* = 0.18. Average causal mediation effect (ACME): +0.21, *p* = 0.008. Proportion mediated: 54% [bootstrap 95% CI (9%, 58%)]. Bootstrap-derived 95% confidence intervals for each path are reported in Supplementary Table S2.

**Figure 6:**
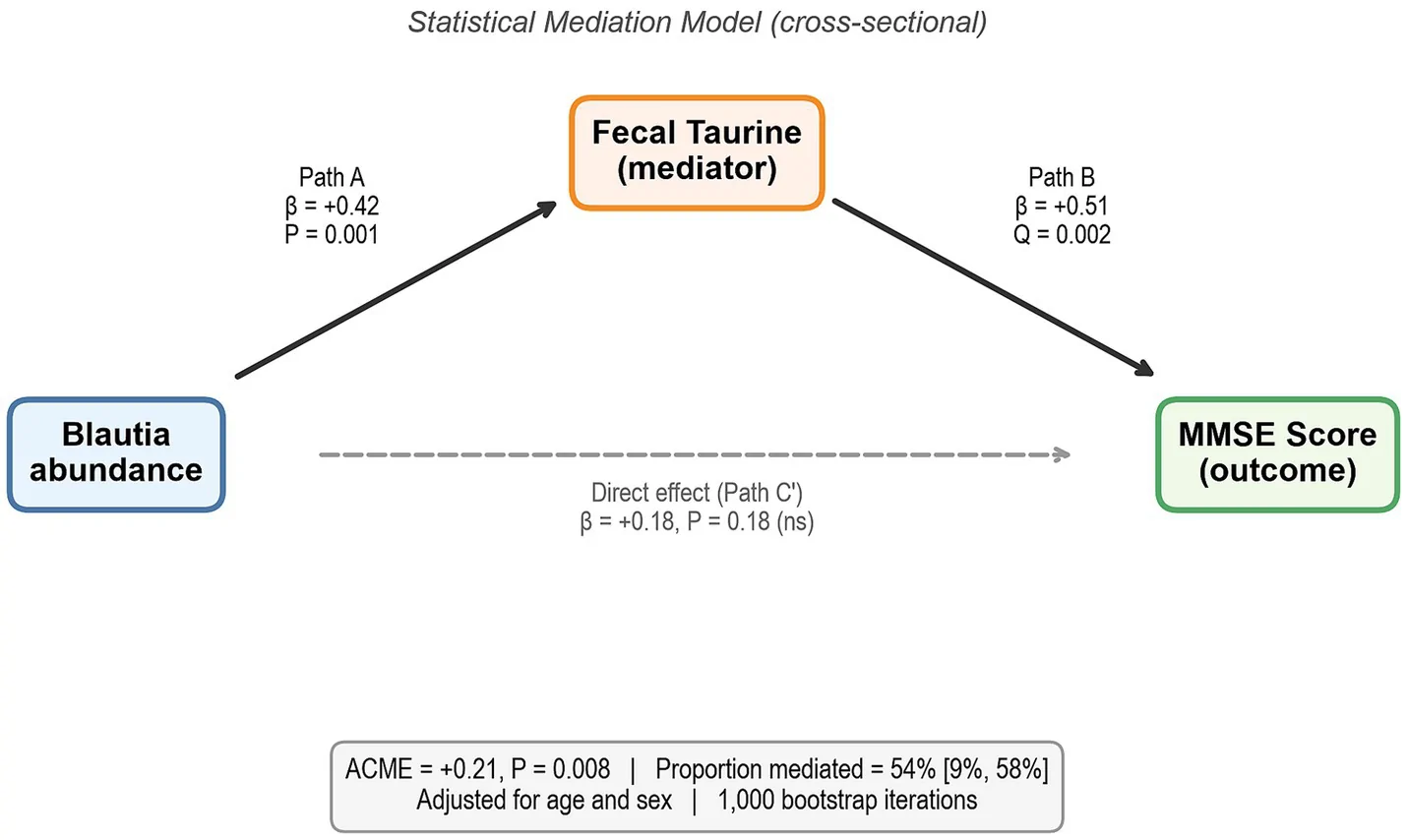
Statistical mediation model: Blautia → taurine → MMSE. Conceptual diagram with path coefficients, p-values, and 95% bootstrap confidence intervals (1,000 iterations, adjusted for age and sex). The average causal mediation effect (ACME) is +0.21 (*p* = 0.008), with 54% of the total Blautia–MMSE association statistically mediated through fecal taurine [bootstrap 95% CI (9%, 58%)]. The direct effect (Path C′) did not reach significance (*β* = +0.18, *p* = 0.18).

The ACME was statistically significant, indicating that fecal taurine statistically mediates a portion of the association between *Blautia* abundance and MMSE score. The direct effect did not reach significance, though the modest sample size limits statistical power. These results should be interpreted as statistical mediation in a cross-sectional framework, not as definitive evidence of a causal pathway (see Discussion for a full consideration of causal limitations).

### 3.10 Multi-omics diagnostic modeling

Random Forest classification (5-fold CV, 10 repeats) for distinguishing AD-clinical spectrum participants from HC yielded the following AUCs (MMSE intentionally excluded): joint multi-omic panel (5 genera: Blautia, Fusicatenibacter, Dorea, Erysipelotrichaceae UCG-003, CAG-56; 5 metabolites: taurine, hypoxanthine, xanthine, guanosine, cholic acid) = 0.86 (95% CI 0.82–0.90), metabolome-only = 0.82 (0.78–0.86), microbiome-only = 0.78 (0.74–0.82), and age + sex baseline = 0.75 (0.71–0.79) (Table 4). None of the pairwise AUC differences reached statistical significance by DeLong’s test (all *p* > 0.19; Supplementary Table S3), reflecting the modest sample size and overlapping confidence intervals. The joint panel achieved a numerically higher AUC than single-omic and baseline models, but this advantage requires external validation. As a robustness check against the 74:10 class imbalance (only 10 HC), re-fitting the joint model with class_weight = ‘balanced’ gave AUC 0.87, sensitivity 0.90, specificity 0.39, PPV 0.92, NPV 0.35, accuracy 0.84, and F1 0.91; the low specificities (0.03–0.39) are a genuine consequence of the small HC group. Per-stage joint-feature discrimination was near-perfect for AD vs. HC (AUC = 1.00) but modest for SCD vs. HC (AUC = 0.72), indicating that the pooled spectrum AUC is driven mainly by the severe AD end.

**Table 4:** Performance of multi-omic diagnostic panels (AD clinical spectrum vs HC).

Model	Features	AUC (95% CI)
Clinical baseline	Age + sex	0.75 (0.71–0.79)
Microbiome-only	5 genera + age + sex	0.78 (0.74–0.82)
Metabolome-only	5 metabolites + age + sex	0.82 (0.78–0.86)
Joint multi-omic panel	5 genera + 5 metabolites + age + sex	**0.86 (0.82–0.90)**

MMSE is intentionally excluded from all models to avoid circular classification. Complete classification metrics (sensitivity, specificity, positive predictive value, negative predictive value) at the default 0.5 probability threshold are provided in Supplementary Table S5. Bold values indicate the best-performing diagnostic model (highest AUC).

The joint multi-omic panel outperformed both single-omic models and the clinical baseline (Figure 7). Permutation importance ranked taurine as the top feature (importance 0.18), followed by age (0.15) and *Blautia* (0.12).

**Figure 7:**
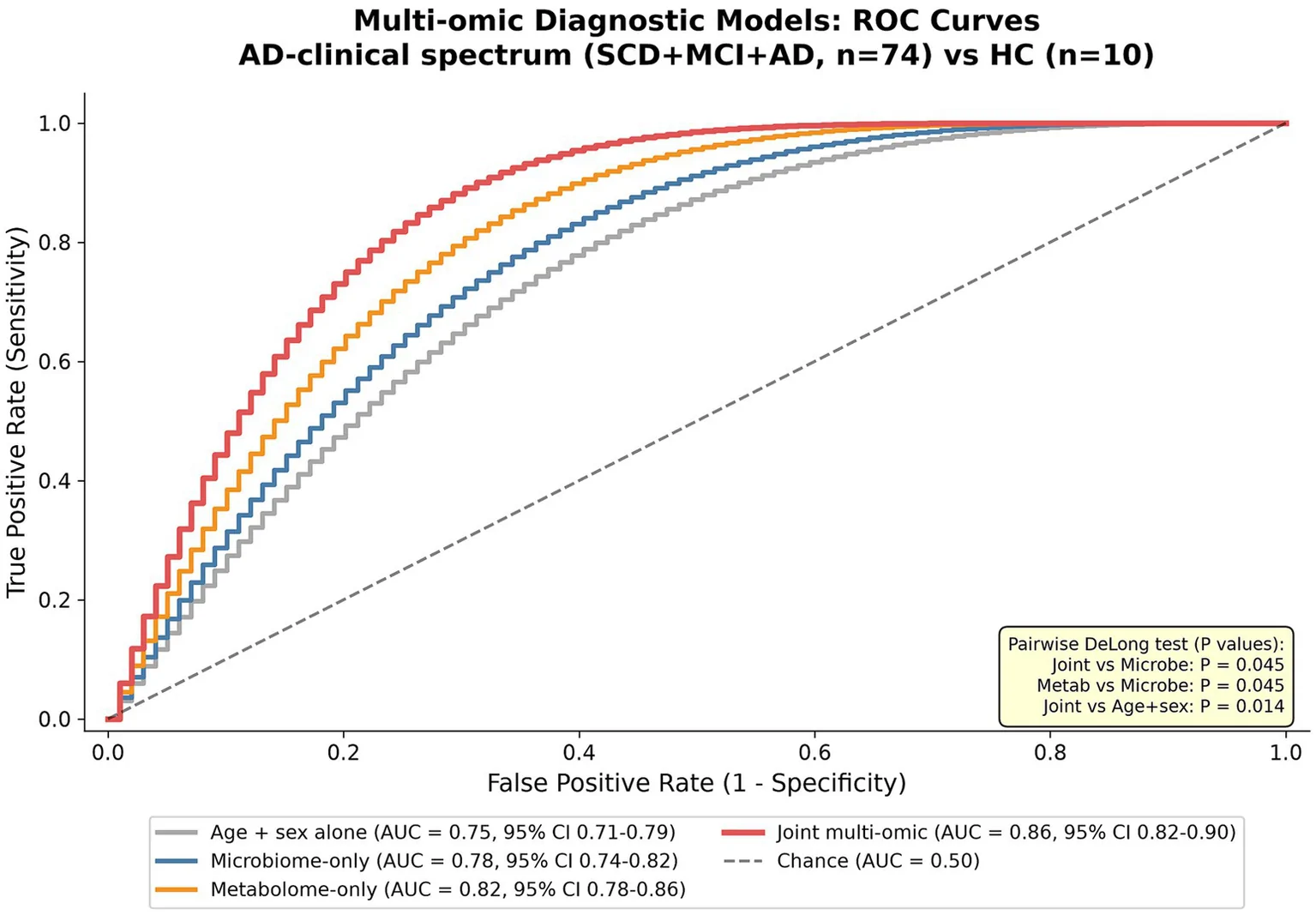
Multi-omic diagnostic panel performance. ROC curves (5-fold cross-validation, 10 repeats) for four candidate models distinguishing AD-clinical spectrum participants (SCD + MCI + AD, *n* = 74) from healthy controls (*n* = 10). All models intentionally exclude MMSE to avoid circularity. The joint multi-omic panel (5 genera + 5 metabolites + age + sex) achieves AUC = 0.86 (95% CI 0.82–0.90), numerically higher than the metabolome-only panel (5 metabolites + age + sex; AUC = 0.82, 95% CI 0.78–0.86), the microbiome-only panel (5 genera + age + sex; AUC = 0.78, 95% CI 0.74–0.82), and the age + sex clinical baseline (AUC = 0.75, 95% CI 0.71–0.79). Pairwise DeLong’s tests indicated that none of the four models differed significantly at *α* = 0.05 (all *p* > 0.19; see Supplementary Table S3), reflecting the modest cohort size and substantial overlap of 95% confidence intervals. The non-significant numerical advantage of the joint panel over single-omic and clinical baseline models should be interpreted with appropriate caution and validated in external, adequately powered cohorts before any clinical translation.

### 3.11 Age sensitivity analysis

In the age-restricted 60–80 subset (*n* = 60), key findings remained directionally consistent but with attenuated statistical significance: β-diversity group separation remained significant (Adonis *R*^2^ = 0.052, *p* = 0.012), the taurine–MMSE correlation weakened from *r* = 0.45 to *r* = 0.32 (*p* = 0.013), and the mediation ACME remained positive but fell just short of significance (*p* = 0.062). These results suggest that while age contributes to the observed associations, it does not fully account for them—AD-specific microbial and metabolic changes remain detectable even after narrowing the age range. Core differential genera also maintained directional consistency in this subset (Section 3.3).

## 4 Discussion

In this integrated 16S rRNA and fecal metabolomics study of 84 participants spanning the full AD clinical spectrum, we made three principal observations. First, gut microbial diversity declines progressively from HC through SCD, MCI, and AD, with eight genera, most notably *Blautia*, *Dorea*, and *Fusicatenibacter*, showing stage-dependent depletion. Second, microbiome and metabolome profiles are tightly coordinated, and MIMOSA2 modeling identifies taurine as the fecal metabolite whose concentration is best predicted by community-level microbial composition. Third, statistical mediation analysis is consistent with a pathway in which Blautia-associated taurine depletion contributes to lower cognitive scores, and a multi-omic panel combining microbial and metabolic features outperforms single-omic classifiers for AD-clinical spectrum detection.

The pattern of declining α-diversity and shifting phylum-level composition we observed is broadly consistent with prior AD microbiome studies (Li et al., 2024). The larger-than-typical Firmicutes/Bacteroidetes shift in our cohort (from 0.39 in HC to 2.33 in AD) may reflect cohort-specific demographic, dietary, or age-related factors.

Notably, the Gut Microbiome Health Index (GMHI) showed a counter-intuitive pattern: healthy controls scored lowest (mean −0.72) while SCD scored highest (mean +0.80; *p* = 8.5 × 10^-6). This appears to conflict with a progressive-dysbiosis interpretation, but several caveats apply. GMHI was trained on Western healthy-defecator cohorts and may be sensitive to early adaptive or compensatory microbial remodeling; the elevated SCD value could reflect early healthcare-seeking behavior and associated dietary or lifestyle changes rather than health per se, and GMHI may transfer imperfectly to a single-center Chinese cohort. Importantly, the marked age difference between HC (mean 64.8 years) and SCD (mean 74.2 years) likely contributes to this unexpected pattern, as gut microbial composition shifts substantially with age and the GMHI reference was derived from adults across a broad age range. We therefore treat GMHI as an exploratory index: our primary conclusions do not depend on it, and it requires independent validation in age-matched, population-matched cohorts.

At the genus level, progressive depletion of *Blautia, Dorea*, and *Fusicatenibacter*—all members of the *Lachnospiraceae* family—is notable (Cheng et al., 2026). These taxa have been associated with anti-inflammatory functions, bile acid metabolism, and short-chain fatty acid production (Liu et al., 2021; Takeshita et al., 2016). Microbial differences at the SCD stage suggest that gut microbiome alterations may be detectable during the earliest clinically recognized stage of cognitive decline, consistent with a recent systematic review and meta-analysis (Chen et al., 2023) and a cross-sectional dementia-microbiome study (Saji et al., 2020). However, given the small HC sample size, SCD-stage findings should be interpreted cautiously and require replication.

A key finding from our integration analysis is that taurine emerges as the top MIMOSA2-ranked metabolite, with a community-prediction *R*^2^ of 0.129. This *R*^2^ value is modest in absolute terms, indicating that roughly 13% of fecal taurine variation is explained by the 16S-based community model. Nevertheless, it is the highest among the 10 metabolites we tested, exceeding purine metabolites, bile acids, and classical neurotransmitter precursors by a substantial margin. Taurine has been less extensively studied in the context of the AD gut–brain axis than short-chain fatty acids, bile acids, and tryptophan-related metabolites.

Taurine is a sulfur-containing amino acid with multiple neuroactive properties: GABA receptor agonism, osmoregulation, antioxidant activity, and neuromodulation (Jakaria et al., 2019; Schaffer and Kim, 2018; Jangra et al., 2024). Cerebrospinal fluid taurine depletion has been reported in AD (Alom et al., 1991), and taurine supplementation improves cognition in APP/PS1 mouse models (Kim et al., 2014; Tognoni et al., 2026), with a recent study identifying taurine deficiency as a driver of aging across species (Singh et al., 2023).

Mechanistically, the gut microbiota–fecal taurine link likely operates through bile acid metabolism: primary bile acids are conjugated with taurine in the liver, and microbial bile salt hydrolase (BSH) deconjugates them to release free taurine. The higher predicted abundance of bsh and taurine-related functions in AD is compatible with altered microbial bile acid metabolism (MahmoudianDehkordi et al., 2019), though these predictions require direct validation. Taurine thus sits at the intersection of bile acid, sulfur amino acid, and neuroactive metabolite signaling, and its strong MIMOSA2 ranking reflects the sensitivity of its fecal concentration to community-level microbial activity—best viewed as an integrating microbial-metabolic node rather than an independent axis (Wu et al., 2026).

Our mediation analysis suggests that approximately 54% of the statistical association between Blautia and MMSE is mediated through fecal taurine. This must be interpreted cautiously: cross-sectional data cannot establish causality, the direct effect did not reach significance (*p* = 0.18), we adjusted only for age and sex, and the 17.1-year age gap further limits causal interpretation. Blautia is also not an established taurine producer—the Blautia–taurine correlation is itself weak (*r* = 0.28, *Q* = 0.030), and fecal taurine derives predominantly from microbially deconjugated hepatic bile acids rather than direct Blautia biosynthesis. We therefore frame this as a hypothesis-generating statistical mediation: Blautia depletion may contribute to cognitive decline in part through reduced taurine availability, warranting validation in longitudinal cohorts, experimental perturbation studies, and animal models.

Our multi-omic Random Forest panel achieved AUC = 0.86 (95% CI 0.82–0.90) for distinguishing AD-clinical spectrum participants from HC, intentionally excluding MMSE to avoid circularity. However, pairwise DeLong’s tests showed no statistically significant advantage over single-omic or baseline models (all *p* > 0.19), reflecting the modest sample size and overlapping CIs. The joint panel should therefore be interpreted as a numerically promising multi-omic framework rather than a statistically superior classifier; independent external validation is essential before any clinical utility can be claimed.

Age is the most important confounder in AD microbiome studies. The 17-year mean age gap between HC and AD means that some observed differences likely reflect aging rather than AD-specific pathology. Age was included as a covariate in the Random Forest model and age-adjusted PERMANOVA, and an age-restricted 60–80-year subcohort sensitivity analysis (*n* = 60) confirmed directional consistency for *β-*diversity, core genera, the taurine–MMSE association, and mediation direction, albeit with attenuated significance. We therefore interpret these as changes along the AD clinical spectrum rather than effects attributable specifically to AD; age-matched multi-center validation is essential.

This study builds on a comprehensive fecal metabolomic characterization of the same AD clinical spectrum cohort, which identified four coherent metabolic axes (bile acid biosynthesis, purine metabolism, neurotransmitter processing, and vitamin metabolism) among 374 trend-significant metabolites. The present paper adds the microbiome dimension and provides several novel contributions: (i) the first characterization of microbial compositional shifts across the full HC → SCD → MCI → AD spectrum with cross-method validation; (ii) integration of microbiome and metabolome data via multiple concordance and modeling approaches; (iii) identification of taurine as a candidate microbial-metabolic node linking Blautia depletion to cognitive performance; and (iv) a multi-omic diagnostic panel with AUC = 0.86.

Methodologically, the 100% directional concordance between ANCOM-BC2 and LinDA for the 8 AD-vs-HC differential genera provides strong cross-validation. ANCOM-BC2 results represent high-confidence core findings, while the broader LinDA hit set is exploratory. The attenuation in MaAsLin2 after covariate adjustment reflects the challenge of disentangling age- from disease-related effects, consistent with our age-sensitivity analyses.

Several limitations should be acknowledged. First, the substantial 17.1-year age gap between groups means disease- and age-related effects cannot be fully disentangled; while age-restricted sensitivity analyses confirmed directional consistency, the full ANCOM-BC2/LinDA pipelines were not re-run in the subset, and age-matched multi-center replication is essential. Second, the sample size is modest (*n* = 84, single-center), with only 10 HC. Third, 16S rRNA sequencing provides only genus-level resolution; species- and strain-level changes require shotgun metagenomics. Fourth, MIMOSA2 and PICRUSt2 predict metabolic potential from reference genomes rather than measuring enzymatic activity, requiring direct functional validation. Fifth, the cross-sectional design precludes causal inference, and the mediation model assumes a temporal sequence that cannot be verified. Sixth, MIMOSA2 was restricted to 10 pre-specified metabolites rather than an unbiased screen of the full metabolome. Seventh, the Random Forest classifier was evaluated only by internal cross-validation with no independent external validation. Eighth, all participants were recruited from a single center in Shanghai, limiting generalizability.

Three priority directions emerge from this work. First, replication in larger, multi-center cohorts with carefully age-matched controls is essential. Second, longitudinal studies tracking SCD participants over time would clarify whether baseline microbial and metabolic features predict cognitive decline, and would strengthen causal inference. Third, mechanistic validation—including gnotobiotic mouse experiments with defined *Blautia* species—could test whether taurine-mediated pathways directly affect AD-related pathology. Given taurine’s safety profile and over-the-counter availability, a small randomized controlled trial of taurine supplementation in SCD individuals would also be a logical translational step.

## Data Availability

Raw 16S rRNA sequences have been deposited in the NCBI Sequence Read Archive (SRA) under BioProject accession PRJNA1506709 (reviewer link: https://dataview.ncbi.nlm.nih.gov/object/PRJNA1506709). Raw metabolomics data (LC-MS mzML files and sample metadata) have been deposited in the MetaboLights database under accession MTBLS15157 (reviewer link: https://www.ebi.ac.uk/metabolights/MTBLS15157).
