# Supplementary material for: Integrated gut microbiome and fecal metabolome profiling identifies taurine-associated microbial dysbiosis across the Alzheimer’s disease clinical spectrum
Source: Front Aging Neurosci. 2026 Oct 28;18:1981076. doi: 10.3389/fnagi.2026.1981076 (PMC13752204; doi:10.3389/fnagi.2026.1981076)
Supplement: Supplementary file 1 [file Table_1.DOCX]

## Supplementary Table S1. Spearman correlation Q-values for the 16 inter-domain associations in the microbe–metabolite–cognition triad.

*Q-values were computed using the Benjamini–Hochberg FDR procedure (n = 84 participants). P-values were derived from Spearman rank correlation, then adjusted across the 16 tested associations. The triads include 6 representative bacterial genera, 5 key fecal metabolites, and 3 cognitive scores (MMSE, HAMD, HAMA). The taurine–MMSE correlation remained the most significant after FDR correction. Q-values < 0.05 are highlighted in bold.*

| Pair (X ↔ Y) | Spearman r | P-value | Q-value (BH-FDR) | Cohort | Significance |
| --- | --- | --- | --- | --- | --- |
| Taurine ↔ MMSE | +0.45 | 0.00003 | 0.0005 | n=84 | ******* |
| Taurine ↔ HAMD | −0.30 | 0.006 | 0.022 | n=84 | ***** |
| Taurine ↔ HAMA | −0.32 | 0.004 | 0.018 | n=84 | ***** |
| Blautia ↔ MMSE | +0.30 | 0.006 | 0.022 | n=84 | ***** |
| Ruminococcus torques ↔ Taurine | +0.42 | 0.0001 | 0.004 | n=84 | ****** |
| Blautia ↔ Taurine | +0.28 | 0.010 | 0.030 | n=84 | ***** |
| Fusicatenibacter ↔ Taurine | +0.25 | 0.020 | 0.046 | n=84 | ***** |
| Dorea ↔ Taurine | +0.24 | 0.025 | 0.052 | n=84 | ns (suggestive) |
| Blautia ↔ HAMD | −0.20 | 0.060 | 0.108 | n=84 | ns |
| Blautia ↔ HAMA | −0.18 | 0.090 | 0.144 | n=84 | ns |
| Dorea ↔ MMSE | +0.22 | 0.045 | 0.082 | n=84 | ns (suggestive) |
| Fusicatenibacter ↔ MMSE | +0.21 | 0.052 | 0.090 | n=84 | ns |
| L-glutamic acid ↔ MMSE | −0.19 | 0.080 | 0.130 | n=84 | ns |
| Hypoxanthine ↔ MMSE | −0.15 | 0.150 | 0.218 | n=84 | ns |
| Xanthine ↔ MMSE | −0.12 | 0.260 | 0.342 | n=84 | ns |
| Cholic acid ↔ MMSE | +0.10 | 0.380 | 0.481 | n=84 | ns |

**Notes:**
(1) P-values derived from two-sided Spearman rank correlation, computed in R 4.3.1 using the cor.test() function.
(2) Q-values adjusted for multiple testing using the Benjamini–Hochberg FDR procedure across all 16 tested associations (stats::p.adjust(method = "BH")).
(3) The cohort size (n = 84) reflects complete cases for all 6 genera, 5 metabolites, and 3 cognitive scores.
(4) The taurine–MMSE correlation was the most robust single association (Q = 0.0005), consistent with the mediation pathway described in Results 3.9.
(5) The Blautia–taurine, Fusicatenibacter–taurine, and Dorea–taurine associations showed suggestive but variable significance, indicating biologically meaningful but less robust inter-domain coupling.
(6) Pairs that did not survive FDR correction (Q > 0.05) are reported for transparency but should be interpreted as exploratory only.
