# Supplementary material for: Integrated gut microbiome and fecal metabolome profiling identifies taurine-associated microbial dysbiosis across the Alzheimer’s disease clinical spectrum
Source: Front Aging Neurosci. 2026 Oct 28;18:1981076. doi: 10.3389/fnagi.2026.1981076 (PMC13752204; doi:10.3389/fnagi.2026.1981076)
Supplement: Supplementary file 2 [file Table_2.DOCX]

## Supplementary Table S2. Statistical mediation model: *Blautia* → fecal taurine → MMSE.

*Path coefficients, bootstrap 95% confidence intervals, and significance levels for the three-path mediation model. Adjusted for age and sex. 1,000 bootstrap iterations. Effective sample size n = 82 (after listwise exclusion of one SCD and one MCI with missing taurine measurements). The mediation model is illustrated in Figure 6.*

| Path | Description | β (path coefficient) | Bootstrap 95% CI | P-value |
| --- | --- | --- | --- | --- |
| A | Blautia → Taurine (independent → mediator) | +0.42 | [+0.21, +0.61] | **< 0.001** |
| B | Taurine → MMSE (mediator → dependent) | +0.51 | [+0.27, +0.74] | **< 0.001** |
| C | Total effect (Blautia → MMSE) | +0.39 | [+0.18, +0.59] | 0.001 |
| C' | Direct effect (Blautia → MMSE, controlling for taurine) | +0.18 | [-0.07, +0.42] | 0.180 |
| ACME | Average Causal Mediation Effect (A × B) | +0.21 | [+0.06, +0.36] | 0.008 |
| ADE | Average Direct Effect (C') | +0.18 | [-0.07, +0.42] | 0.180 |
| Proportion mediated | (ACME) / (ACME + ADE) × 100% | 54% | [9%, 58%] | 0.012 |

**Notes:
(1) The mediation model was fitted on the full cohort after listwise exclusion of missing values; effective n = 82 (one SCD and one MCI had missing taurine measurements).
(2) The model was adjusted for age and sex. This provides partial protection against the 17.1-year age gap between AD and HC groups, although residual confounding by other age-related factors (diet, polypharmacy, residential status) cannot be fully excluded.
(3) Bootstrap-derived 95% CIs were obtained from 1,000 nonparametric bootstrap iterations using the *mediation* R package v4.5.0 (Tingley et al., 2014).
(4) The ACME (Average Causal Mediation Effect) is statistically significant (P = 0.008), supporting a partial mediation pathway from *Blautia* through fecal taurine to MMSE.
(5) The direct effect (C') is non-significant (P = 0.180), suggesting that the indirect (mediation) pathway accounts for the majority of the *Blautia*–MMSE association.
(6) The proportion mediated (54%) is consistent with substantial — but not complete — mediation by taurine. The remaining 46% of the effect may be mediated by other microbial or metabolic pathways not captured in this model.
(7) Sensitivity analysis using an age-restricted 60–80 year subset (n = 60) yielded directionally consistent results: ACME = +0.18, P = 0.024; ADE = +0.16, P = 0.230; proportion mediated = 32%.**
