# Supplementary material for: Integrated gut microbiome and fecal metabolome profiling identifies taurine-associated microbial dysbiosis across the Alzheimer’s disease clinical spectrum
Source: Front Aging Neurosci. 2026 Oct 28;18:1981076. doi: 10.3389/fnagi.2026.1981076 (PMC13752204; doi:10.3389/fnagi.2026.1981076)
Supplement: Supplementary file 3 [file Table_3.DOCX]

## Supplementary Table S3. DeLong's test for pairwise AUC comparison across the four diagnostic models.

*Hanley–McNeil approximation, n_pos = 74 (SCD + MCI + AD), n_neg = 10 (HC).* SE(AUC) computed from Hanley–McNeil (1982) formula. z-statistic calculated for independent AUCs. None of the six pairwise comparisons reached statistical significance at α = 0.05, reflecting the modest cohort size and substantial overlap of 95% confidence intervals.

| Model pair | AUC1 | AUC2 | ΔAUC | z-statistic | P-value |
| --- | --- | --- | --- | --- | --- |
| Joint multi-omic vs Metabolome-only | 0.86 | 0.82 | +0.04 | 0.538 | 0.591 |
| Joint multi-omic vs Microbiome-only | 0.86 | 0.78 | +0.08 | 0.992 | 0.321 |
| Joint multi-omic vs Age + sex alone | 0.86 | 0.75 | +0.11 | 1.294 | 0.196 |
| Metabolome-only vs Microbiome-only | 0.82 | 0.78 | +0.04 | 0.464 | 0.643 |
| Metabolome-only vs Age + sex alone | 0.82 | 0.75 | +0.07 | 0.775 | 0.438 |
| Microbiome-only vs Age + sex alone | 0.78 | 0.75 | +0.03 | 0.314 | 0.754 |
|  |  |  |  |  |  |

**Notes:**
(1) DeLong, DeLong, and Clarke-Pearson (1988) "Comparing the areas under two or more correlated ROC curves: a nonparametric approach", Biometrics 44:837-845.
(2) Hanley and McNeil (1982) "The meaning and use of the area under a receiver operating characteristic (ROC) curve", Radiology 143:29-36.
(3) The 95% CIs used for SE estimation were obtained from 2,000 bootstrap replicates of the 5-fold cross-validated Random Forest predictions (Methods 2.10).
(4) Because the four models are not perfectly correlated (different feature sets and different random forest seeds), an independent z-test is used as a conservative approximation of the paired DeLong's test. The true paired DeLong's test may yield slightly smaller P-values if correlations between models are strong.
(5) n_pos = 74 corresponds to the AD-continuum group (SCD n=22 + MCI n=23 + AD n=29); n_neg = 10 corresponds to the healthy control (HC) group.
(6) Interpretation: none of the four models differ significantly at α = 0.05. The joint multi-omic panel achieves a numerically higher AUC than the three single-feature-set models, but this advantage is not statistically robust in the current cohort. External validation in larger, multi-center cohorts is required before any clinical translation.
